# Cardiac mortality benefit of direct admission to percutaneous coronary intervention–capable hospital in acute myocardial infarction: Community registry–based study

**DOI:** 10.1097/MD.0000000000025058

**Published:** 2021-03-12

**Authors:** Min Gyu Kang, Yoomee Kang, Kyehwan Kim, Hyun Woong Park, Jin-Sin Koh, Jeong Rang Park, Seok-Jae Hwang, Jong-Hwa Ahn, Yongwhi Park, Young-Hoon Jeong, Choong Hwan Kwak, Jin-Yong Hwang

**Affiliations:** a Department of Internal Medicine, Gyeongsang National University School of Medicine and Gyeongsang National University Hospital, Jinju; b Department of Internal Medicine, Gyeongsang National University School of Medicine and Cardiovascular Center, Gyeongsang National University Changwon Hospital, Changwon, Republic of Korea.

**Keywords:** acute myocardial infarction, cardiac mortality, direct admission, percutaneous coronary intervention, percutaneous coronary intervention–capable hospital

## Abstract

Appropriate risk stratification and timely revascularization of acute myocardial infarction (AMI) are available in percutaneous coronary intervention (PCI) – capable hospitals (PCHs). This study evaluated whether direct admission vs inter-hospital transfer influences cardiac mortality in patients with AMI. This study was conducted in the PCH where the patients were able to arrive within an hour. The inclusion criteria were AMI with a symptom onset time within 24 hours and having undergone PCI within 24 hours after admission. The cumulative incidence of cardiac death after percutaneous coronary intervention was evaluated in the direct admission versus inter-hospital transfer groups. Among the 3178 patients, 2165 (68.1%) were admitted via inter-hospital transfer. Patients with ST-segment elevation myocardial infarction (STEMI) in the direct admission group had a reduced symptom onset-to-balloon time (121 minutes, *P* < .001). With a median period of 28.4 (interquartile range, 12.0–45.6) months, the cumulative incidence of 2-year cardiac death was lower in the direct admission group (NSTEMI, 9.0% vs 11.0%, *P* = .136; STEMI, 9.7% vs 13.7%, *P* = .040; AMI, 9.3% vs 12.3%, *P* = .014, respectively). After the adjustment for clinical variables, inter-hospital transfer was the determinant of cardiac death (hazard ratio, 1.59; 95% confidence interval, 1.08–2.33; *P* = .016). Direct PCH admission should be recommended for patients with suspected AMI and could be a target for reducing cardiac mortality.

## 1 Introduction

To reduce cardiac death and heart failure in acute myocardial infarction (AMI), appropriate risk stratification and timely percutaneous coronary intervention (PCI) are a critical point of care in early management.^[1–3]^ In ST-segment elevation myocardial infarction (STEMI), critical pathways to reduce ischemia time have been developed and performed both in and out of hospitals, strongly contributing to myocardial salvage.^[4,5]^ STEMI guidelines recommend that physicians in PCI-non-capable hospitals (PNCHs) make decisions to transfer patients to PCI-capable hospitals (PCHs) or those offering onsite fibrinolysis by anticipating the first medical contact-to-device time (FDT) of 120 minutes.

With an increase in PCH density and the development of the emergency medical system, it became possible to access the PCH within an hour in the majority of cases.^[6]^ Because timely PCI leads to better clinical outcomes, most AMI regional networks prefer direct admission to a PCH.^[7]^ However, optimal symptom onset-to-balloon time (SBT) is not being achieved for the considerable patients with STEMI.^[8]^ Recent studies reported that direct admission to a PCH has a beneficial influence on cardiac mortality in STEMI patients.^[9,10]^ And, direct admission to a PCH can be an important determinant of better clinical outcomes in cases of non-STEMI (NSTEMI), especially in high-risk patients.^[11,12]^

There has been little evidence of direct PCH admission as a determinant of cardiac death in real world data of all-comer AMI. Accordingly, this study aimed to determine whether direct admission to PCH vs inter-hospital transfer benefits cardiac mortality in patients with AMI using current community registry–based data.

## 2 Methods

### 2.1 Study population

**Figure 1:**
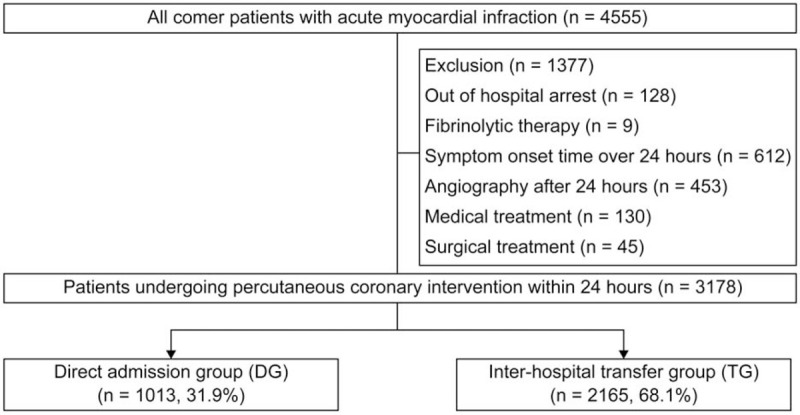
Study flow chart.

We analyzed registry data between January 2010 and December 2017 from Gyeongsang National University Hospital (GNUH), the only PCH in the Southwestern area of Gyeongnam Province, South Korea. The GNUH-AMI registry database was established by the National Gyeongnam Regional Cardiovascular Center. AMI was defined as an elevation of cardiac biomarkers with ischemic symptoms or changes on electrocardiography (ECG), imaging evidence of recent loss of viable myocardium, or a new regional wall motion abnormality. The presence of ST-segment elevation on a standard 12-lead surface ECG was determined at the time of the emergency department visit. ST-segment elevation was defined as an increase in the ST-segment >2 mm in ≥2 precordial leads or >1 mm in ≥2 limb leads on standard 12-lead ECG. Management including risk stratification was carried out according to current guideline of NSTEMI.^[11]^ We included all patients with AMI who had a symptom onset time within 24 hours and were treated with PCI within 24 hours after admission (Fig. 1). We excluded patients with out-of-hospital cardiac arrest, fibrinolytic therapy, non-cardiac disease with a life-expectancy <1 year, medical treatment, and surgical treatment. Finally, 3178 patients who met the inclusion criteria were categorized into the direct admission group (DG) and inter-hospital transfer group (TG). Data were collected by a well-trained study coordinator using a standardized case report form and following protocol. Institutional review board approval was obtained from the local ethics committee (No. GNUH 2018–12-024), which waived the need for informed consent. The study was performed in accordance with the Good Clinical Practice Guidelines and the principles of the Declaration of Helsinki.

### 2.2 Percutaneous coronary intervention

All eligible patients underwent PCI as coronary reperfusion therapy. Invasive coronary angiography was performed using a standard technique, and significant coronary artery disease was diagnosed visually if ≥50% luminal diameter narrowing was present in a major epicardial coronary artery. Left main trunk disease was considered as a two-vessel disease, while the presence of more than 2 significant coronary artery diseases was considered multi-vessel disease. A complex lesion was defined as a type B2/C lesion according to the American College of Cardiology (ACC)/American Heart Association (AHA) lesion classification.^[13]^ PCI methods were decided by the operators’ preference, and all procedures were consistent with the current guideline recommendations. Multi-vessel PCI was defined as PCI performed in 2 or more coronary arteries. Primary PCI was performed in the presence of ST-segment elevation at admission, while door-to-balloon time (DBT) was defined as the time from hospital arrival to vessel recanalization by the first balloon inflation or aspiration thrombectomy. In patients with NSTEMI, the revascularization therapy timing was decided using patient risk stratification by interventional cardiologists.

### 2.3 Clinical outcomes

Follow-up clinical data were obtained by independent experienced clinical research coordinators from the patients’ hospital medical records, periodic patient examinations in outpatient clinics, and telephone interviews. We assessed the cumulative incidence of cardiac death after PCI. In the present study, the confirmation of cardiac death required the documentation of significant arrhythmia or cardiac arrest, death attributable to congestive heart failure, or myocardial infarction in the absence of other precipitating factors.

### 2.4 Statistical analysis

Continuous variables are presented as mean ± standard deviation if distributed symmetrically or with mild skew and compared using one-way analysis of variance (ANOVA). Other skewed continuous variables are summarized as median (interquartile range) and were compared using the Kruskal–Wallis rank sum test. The Chi-Squared test or Fisher exact test was used to determine the significance of the differences in categorical variables. Comparisons of continuous data among groups were conducted by ANOVA with post-hoc analysis. A general linear model of univariate analysis was applied to quantify the N-terminal of the prohormone brain natriuretic peptide (NT-pro BNP) level. Variables with a univariate association with cardiac death were entered stepwise into a logistic model. A multivariate logistic regression analysis was conducted to identify the contributing factors of cardiac death minutes among variables with values of *P* < .01 in the univariate analysis. The survival analysis of clinical outcomes was performed using the Kaplan–Meier curve. *P* values <.05 were considered statistically significant. Analyses were performed using the Statistical Package for Social Sciences version 21.0 (SPSS Inc., Chicago, IL).

## 3 Results

### 3.1 Baseline characteristics

Among the 3178 patients, 2165 (68.1%) were admitted via inter-hospital transfer. The TG was older and had a male predominance (Table 1). Current smoking was more prevalent in the TG. The DG had a higher proportion of patients with previous PCI experience. The proportions of STEMI and cardiogenic shock were similar between the 2 groups. There were no intergroup differences in coronary angiographic findings. The mean NT-pro BNP level was higher in the TG (2279 ± 4993 vs 1573 ± 3943 pg/ml, *P* < .001). And, left ventricular ejection fraction (LVEF) was better in the DG (54 ± 9% vs 52 ± 9%, *P* < .001). Discharge medications were balanced between the 2 groups.

**Table 1:** Patients’ baseline characteristics.

Variable	Inter-hospital transfer (n = 2165)	Direct admission (n = 1013)	*P* value
Clinical characteristic			
Age, years	66 ± 12	64 ± 11	<.001
Female sex	291 (25.4)	859 (34.0)	<.001
Hypertension	1059 (48.9)	462 (45.7)	.086
Diabetes	598 (27.6)	271 (26.8)	.620
Dyslipidemia	128 (11.2)	257 (10.2)	.383
Current smoking	958 (44.2)	107 (40.2)	.031
Previous PCI	162 (7.5)	185 (18.3)	<.001
Ischemic stroke	142 (6.5)	64 (6.3)	.737
Chronic kidney disease	347 (16.0)	171 (16.9)	.537
Clinical presentation			
ST-segment elevation	956 (44.2)	460 (45.4)	.508
Systolic BP, mmHg	132 ± 28	133 ± 30	.141
Heart rate, beats per minute	76 ± 20	75 ± 21	.110
Cardiogenic shock	193 (9.0)	95 (9.5)	.595
Coronary angiographic and procedural characteristics			
Infarct related artery			.588
Left main	76 (3.5)	39 (3.8)	
Left anterior descending	820 (37.8)	406 (40.0)	
Right coronary artery	760 (35.2)	355 (35.0)	
Left circumflex	509 (23.5)	213 (21.0)	
Complex lesion^∗^	1870 (86.3)	889 (87.7)	.207
Multi-vessel disease	1077 (49.7)	502 (49.5)	.946
Multi-vessel PCI	670 (30.9)	316 (31.7)	.489
Laboratory findings			
WBC count, ×10^3^/mm^3^	10.3 ± 4.9	10.5 ± 4.1	.060
Hemoglobin, g/dl	12.9 ± 1.8	12.8 ± 1.9	.520
Creatinine, mg/dl	1.1 ± 0.9	1.0 ± 0.9	.068
NT-pro BNP, pg/ml	2279 ± 4993	1573 ± 3943	<.001
LVEF, %	52 ± 9	54 ± 9	<.001
Discharge medication			
Aspirin	2006 (98.9)	951 (99.0)	.997
Clopidogrel	1521 (75.0)	705 (72.5)	.121
Prasugrel	59 (2.9)	32 (3.3)	.198
Ticagrelor	422 (20.8)	229 (23.9)	.119
Beta-blocker	1560 (76.9)	723 (75.3)	.304
Angiotensin blocker	1477 (72.8)	702 (73.1)	.708
Statin	1885 (92.9)	882 (91.8)	.262

Data are expressed as number (%) or mean ± standard deviation as appropriate.

∗ Complex lesion was defined as type B2/C lesion according to the American College of Cardiology/American Heart Association lesion classification. BP = blood pressure, LVEF = left ventricle ejection fraction, NT-pro BNP = N-terminal of the prohormone brain natriuretic peptide, PCI = percutaneous coronary intervention, WBC = white blood cell.

### 3.2 In- and out-of-hospital time in STEMI

**Figure 2:**
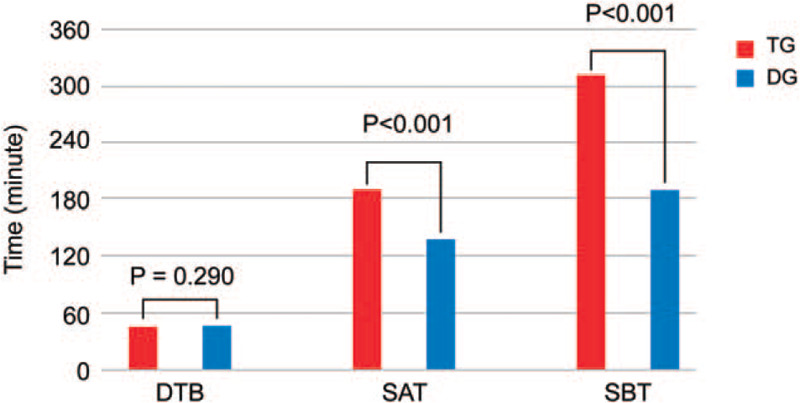
Time in ST-segment elevation myocardial infarction. DBT = door-to-balloon time, SAT = symptom onset-to-admission time, SBT = symptom onset-to-balloon time.

There were total of 1416 (44.6%) STEMI patients; in- and out-of-hospital times were documented (Fig. 2). Median symptom onset-to-admission time (SAT) was shorter by 52 minutes and median SBT was shorter by 121 minutes in the DG vs TG (SAT, 139 minutes vs 191 minutes, *P* < .001; SBT, 312 minutes vs 191 minutes, *P* < .001, respectively). The proportions of patients with an SBT less than 120, 180, and 360 minutes was highly achieved in the DG (SBT < 120 minutes, 46.3% vs 13.5%, *P* < .001; SBT < 180 minutes, 65.7% vs 34.2%, *P* < .001; SBT < 360 minutes, 76.8% vs 64.3%, *P* = .009, respectively).

### 3.3 NT-pro BNP level and LVEF at 30 days

**Figure 3:**
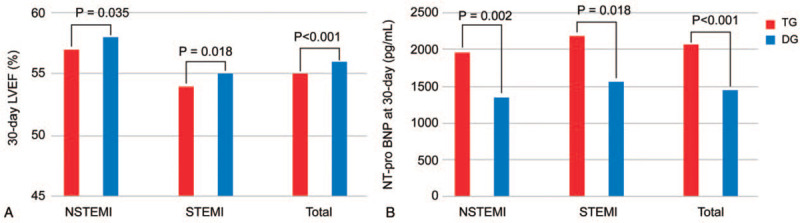
Measurements at 30 days. (A) LVEF. (B) NT-pro BNP. LVEF = left ventricular ejection fraction, NSTEMI = non-ST-segment elevation myocardial infarction, NT-pro BNP = N-terminal of the prohormone brain natriuretic peptide, STEMI = ST-segment elevation myocardial infarction.

The 30-day LVEF and NT-pro BNP level, powerful prognostic factors in AMI, were assessed among survivors (Fig. 3). The 30-day LVEF was higher in the DG than in the TG (NSTEMI, 58 ± 10% vs 57 ± 10%, *P* = .035; STEMI, 55 ± 8% vs 54 ± 9%, *P* = .018; total, 56 ± 7% vs 55 ± 8%, *P* < .001, respectively). The 30-day NT-pro BNP level adjusted by the general linear model for adjusted covariates (age, sex, hematocrit, and renal function) was significantly lower in the DG (NSTEMI, 1348 ± 2735 vs 1961 ± 4705 pg/ml, *P* = .002; STEMI, 1566 ± 3397 vs 2196 ± 4223 pg/ml, *P* = .018; total, 1449 ± 3039 vs 2069 ± 4489 pg/ml, *P* < .001, respectively).

### 3.4 Cardiac mortality

**Figure 4:**
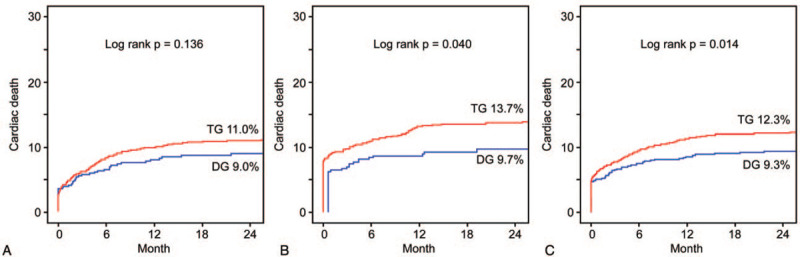
Cumulative incidence of cardiac death. (A) Non-ST-segment elevation myocardial infarction. (B) ST-segment elevation myocardial infarction. (C) Total population of patients with acute myocardial infarction. DG = direct admission group, TG = inter-hospital transfer group.

This study aimed to evaluate the influence of direct PCH admission on in-hospital and long-term cardiac death and whether it contributes to a delay in myocardial revascularization. With a median hospital stay of 4 [3–6] days, in-hospital mortality was significantly lower in the DG (5.7% vs 7.3%; odds ratio, 1.31; *P* = .037). Over a median 28.4 [12.0–45.6] months, the cumulative incidence of 2-year cardiac death was significantly higher in the TG (12.3% vs 9.3%, *P* = .014) (Fig. 4). STEMI patients in the DG showed a significantly lower incidence of 2-year cardiac death than those in the TG (9.7% vs 13.7%, *P* = .040). However, there was no significant intergroup difference in cardiac mortality among NSTEMI patients (9.0% vs 11.0%, *P* = .136).

In the total population of AMI, old age (>65 years), chronic kidney disease, current smoking, multi-vessel disease, reduced LVEF (<50%), and inter-hospital transfer were determinants of cardiac death (Table 2). In this study, inter-hospital transfer was an independent risk factor for death (hazard ratio, 1.59; 95% confidence interval, 1.08–2.33; *P* = .016).

**Table 2:** Determinants of cardiac death in acute myocardial infarction.

	Univariate analysis		Multivariate analysis	
Variable	Hazard ratio (95% CI)	*P* value	Hazard ratio (95% CI)	*P* value
Age, >65 years	3.66 (3.22–4.83)	<.001	2.63 (1.80–3.83)	<.001
Sex, female	2.04 (1.61–2.59)	<.001	1.29 (0.93–1.78)	.118
Diabetes	1.54 (1.20–1.97)	.001	1.16 (0.84–1.58)	.366
Dyslipidemia	1.35 (1.07–1.71)	.011	1.13 (0.84–1.52)	.401
CKD	5.37 (4.20–6.89)	<.001	2.64 (1.92–3.63)	<.001
Current smoking	1.64 (1.28–2.10)	<.001	1.14 (0.82–1.59)	.413
STEMI	1.62 (1.28–2.05)	<.001	1.30 (0.96–1.75)	.080
Cardiogenic shock	3.25 (2.38–4.42)	<.001	1.48 (0.97–2.25)	.067
Multi-vessel disease	1.95 (1.51–2.51)	<.001	1.66 (1.22–2.26)	.001
LVEF <50%	3.45 (2.64–4.50)	<.001	3.57 (2.00–1.75)	<.001
Inter-hospital transfer	1.30 (1.01–1.684)	.045	1.59 (1.08–2.33)	.016

CI = confidence interval, CKD = chronic kidney disease, LVEF = left ventricle ejection fraction, STEMI = ST-segment elevation myocardial infarction.

## 4 Discussion

This analysis is the first to show differences in cardiac mortality of AMI, including NSTEMI, between direct PCH admission and inter-hospital transfer. The main findings of this study are as follows:

1. more than two-thirds (68.1%) of AMI patients were referred to PCH by inter-hospital transfer, although the included patients were able to access the PCH within an hour;
2. in STEMI, direct admission achieved a shorter SBT by 121 minutes and a greater proportion of SBT < 180 minutes;
3. baseline/30-day LVEF and NT-pro BNP level were more favorable in the DG; and
4. direct admission was associated with significantly better in-hospital and long-term cardiac mortality in the total AMI population.

Although the STEMI guideline recommends immediate transfer to a PCH if the physician anticipates an FDT < 120 minutes,^[1,5]^ ideally all patients have a chance of direct PCH admission. In the United States, 79% of the population live in the region with a short driving time of <60 minutes from a PCH, and 34% of patients who visit a PNCH live in regions with very short driving times of <30 minutes.^[8]^ However, more than one-third of STEMI patients in the region with an hour driving time fail to achieve an FDT <120 minutes.^[14,15]^ Our study reported that 68% of patients with AMI experienced inter-hospital transfer in the region with a short driving time of <60 minutes in South Korea. These real-world data suggest that it will be necessary to increase the rate of direct PCH admissions in the regional AMI networks.

Direct admission can decrease ongoing myocardial necrosis and reduce the risk with fibrinolysis therapy, leading to reduced cardiac mortality among patients with AMI.^[16–18]^ However, the results of previous clinical trials showed equal cardiac mortality rates between direct PCH admission and inter-hospital transfer patients who underwent primary PCI within 120 minutes.^[6,19–21]^ Other real-world data also reported similar results in cardiac mortality despite the shortened FDT.^[22,23]^ In contrast, our results suggested the following. First, our study results show a longer SBT difference (121 minutes) than earlier studies. A previous study of Korea Acute Myocardial Infarction Registry showed that the difference in median SBT between the 2 groups was 78 minutes. Although we were unable to define door-in-door-out (DIDO) time, it might have influenced SAT in the region with a short driving time.^[14,24]^ Second, the optimal goal of SBT of STEMI might require a stricter value. The stratification of SBT <120, <180, and <360 minutes and setting a goal could strongly impact survival in patients with AMI.^[25,26]^

This study's findings correspond with those of recent studies, Japanese data and the Polish registry of Acute Coronary Syndrome (PL-ACS).^[9,10]^ The PL-ACS results showed that direct admission was associated with a shorter SAT (44 minutes) and lower mortality rates at 1, 6, and 12 months. In-hospital mortality was paradoxically higher in direct PCH admission, which was explained as selection bias by the authors. Survivors before PCH among the inter-hospital transfers were enrolled in the PL-ACS analysis. In our study, patients with out-of-hospital arrest were excluded and worse cardiac mortality was consistently observed during the hospital stay and long-term follow-up. The Japanese Kamakura Hospital data had a similar time and survival benefit to those of direct admission in our study. Yoichi et al reported the intergroup SBT difference (90 minutes) and that the proportion of SBT <180 minutes was significantly greater in the DG, which might explain the survival benefit. Myocardial salvage via SBT shortening in STEMI demonstrated favorable results of baseline/30-day LVEF and NT-pro BNP levels in the DG.^[27,28]^

In the contemporary management of NSTEMI, a routine invasive strategy has been preferred as a selective invasive strategy because it improves clinical outcomes and reduces recurrent myocardial infarction, heart failure requiring hospitalization, and revascularization.^[11,29]^ The timing of PCI for NSTEMI is dependent on risk stratification because the benefit of an early invasive strategy is evident in high-risk patients.^[30–32]^ If patients with NSTEMI present at a PNCH, current guidelines recommend that very high-risk patients be immediately transferred to a PCH. The early transfer and invasive treatment of high-risk patients with NSTEMI may impact the cardiac survival benefit. However, the Early Glycoprotein IIb/IIIa Inhibition in Patients With Non-ST-segment Elevation Acute Coronary Syndrome (EARLY-ACS) trial showed that patients transferred early to a tertiary hospital experienced a significant time delay from symptom onset to treatment.^[33]^ The Can Rapid Risk Stratification of Unstable Angina Patients Suppress Adverse Outcomes with Early Implementation of the ACC/AHA Guidelines (CRUSADE) trial reported that only 20% of NSTE ACS patients were transferred early (within 48 hours) and lower-risk patients appear to be preferentially transferred early.^[12]^ In this study, PNCHs followed the strategy of rapid transfer in cases of suspicious NSTE ACS irrespective of risk stratification. The TG in this study might have included NSTEMI with a lower or intermediate risk among the TG who did not influence cardiac mortality. Hence, this study did not demonstrate the effect of direct admission on the survival benefit in NSTEMI, consistent with the results of the EARLY-ACS trial. However, even in NSTEMI cases, we first reported that direct admission resulted in greater salvage of the myocardium, representing lower baseline/30-day NT-pro BNP levels and a higher baseline/30-day LVEF. A well-controlled prospective study of these issues is required in the future.

Our study has several limitations. First, the current study was a single-center experience, which may limit its generalization. Selection bias might have been present since it was a community registry-based study. We excluded the out of hospital arrest and fibrinolytic therapy. The consideration of the community situation would be required in the interpretation of our results. Future study with stratified analysis of large sample population will be better reflect which method would be suitable for the 2 methods, such as diabetes mellitus, heart failure, and complex coronary anatomy. Second, we defined the inclusion criteria of SBT <24 hours and PCI within 24 hours, which may have substantial limitations for interpreting the impact of longer delays resulting from inter-hospital transfer. However, because the PNCHs in this study adopted the rapid transfer of patients with suspected NSTE ACS, the TG may rarely have patients who are beyond 24 hours after symptom onset. Third, we could not provide information on socioeconomic status, family relationships, and transport system affecting inter-hospital transfer, and time values including symptoms to FMC time, DIDO time, and transportation time in the TG.

In conclusion, this study demonstrated that direct PCH admission results in greater myocardial salvage effects and short- and long-term survival benefits in patients with AMI. These findings are more evident in patients with STEMI and included reduced total ischemic time in the region in which patients can access PCH within an hour. For survival, direct admission to PCH should be more commonly recommended for patients with suspected AMI.

## Author contributions

**Conceptualization:** Yoomee Kang, Jin-Yong Hwang.

**Data curation:** Kyehwan Kim, Hyun Woong Park, Jin-Sin Koh.

**Formal analysis:** Jong-Hwa Ahn, Yongwhi Park, Young-Hoon Jeong, Choong Hwan Kwak.

**Methodology:** Jeong Rang Park, Seok-Jae Hwang.

**Project administration:** Min Gyu Kang, Yoomee Kang.

**Supervision:** Jin-Yong Hwang.

**Writing – original draft:** Min Gyu Kang.

**Writing – review & editing:** Min Gyu Kang.
